# Beyond the smile: a systematic review of diagnostic tools for peripheral facial paralysis

**DOI:** 10.1007/s13760-024-02630-w

**Published:** 2024-08-29

**Authors:** Roberto Tedeschi, Danilo Donati, Federica Giorgi

**Affiliations:** 1 Department of Biomedical and Neuromotor Sciences (DIBINEM), Alma Mater Studiorum University of Bologna, Via Zamboni 33, 40126 Bologna, Italy; 2 Physical Therapy and Rehabilitation Unit, Policlinico di Modena, Modena, Italy; 3 Clinical and Experimental Medicine PhD Program, University of Modena and Reggio Emilia, Modena, Italy; 4 Pediatric Physical Medicine and Rehabilitation Unit, IRCCS Institute of Neurological Sciences of Bologna, Via Altura 3, Bologna, Italy, IRCCS Institute of Neurological Sciences of Bologna, Via Altura 3, Bologna, Italy

**Keywords:** Peripheral facial paralysis, Rehabilitation, Assessment tools, Facial disability, Synkinesis, ICF framework

## Abstract

**Background:**

Effective rehabilitation of peripheral facial paralysis (PFP) requires reliable assessment tools. This systematic review aimed to identify and validate instruments used in PFP rehabilitation, categorizing them according to the ICF framework.

**Methods:**

A comprehensive search was conducted across PubMed, Cinahl, Web of Science, and Scopus up to April 2024. Observational analytical studies and one non-randomized controlled trial that validated tools for assessing PFP were included.

**Results:**

Thirty-three studies were included, covering twenty different tools. Seventeen tools were related to the "Structure and Function" domain, while three addressed "Activity and Participation." The Sunnybrook and House-Brackmann scales were the most extensively studied. The Sunnybrook scale exhibited excellent intra- and inter-rater reproducibility and internal validity, making it suitable for clinical use. The House-Brackmann scale was user-friendly but had limitations in reproducibility and sensitivity to subtle differences, which newer versions like the FNGS 2.0 aimed to address. The FAME scale showed promise by reducing subjective scoring. Computerized tools, such as eFACE and A-FPG, and instruments for lip asymmetry and ocular involvement demonstrated potential but require further validation. The Facial Disability Index and the FaCE Scale were validated for assessing disability and participation restrictions.

**Conclusion:**

This review identified several validated tools for PFP assessment, with the Sunnybrook and House-Brackmann scales being the most reliable. While emerging tools and computerized programs show promise, they need further validation for routine clinical use. Integrating validated tools into clinical practice is essential for comprehensive assessment and effective rehabilitation of PFP.

## Introduction

The facial nerve, or the seventh cranial nerve, is a complex mixed nerve with motor, sensory, and parasympathetic functions [1, 2]. Predominantly motor, it innervates facial muscles, including the stapedius muscle, provides taste sensation to the anterior two-thirds of the tongue, and controls secretions from the lacrimal, nasal, palatal, submandibular, and sublingual glands [3]. Peripheral facial palsy (PFP) can result from various etiologies, including trauma, infections, and idiopathic causes like Bell’s palsy, which constitutes approximately 70% of PFP cases [4, 5]. This condition significantly affects facial expressions, communication, and overall quality of life, often leading to psychological issues such as depression and anxiety [6]. PFP is characterized by complete paralysis of the facial muscles on the affected side, resulting in asymmetry at rest and pronounced asymmetry during facial movements. Depending on the lesion’s location, sensory and vegetative functions may also be impaired, including reduced taste sensation and decreased salivary and lacrimal secretions [7–9]. Differential diagnosis from central facial palsy, which affects only the lower half of the face contralaterally, is crucial due to distinct etiologies and treatment approaches [10–12]. The complex anatomy of the facial nerve and diverse causes of its dysfunction underscore the need for robust and reliable assessment tools to evaluate the extent of damage and guide treatment. Various assessment scales exist, but there is no universally accepted gold standard, leading to inconsistent use and varying degrees of validation and clinical applicability [13, 14]. Treatment approaches for PFP include conservative and surgical options. Conservative management primarily involves physiotherapy to educate patients, mobilize soft tissues, retrain facial muscles, and manage synkinesis—unintentional movements that occur during voluntary facial movements—if present [8, 15–20]. In acute phases, protecting the eye and preventing complications like corneal ulcers due to lagophthalmos are critical. Pharmacological interventions, including corticosteroids, are recommended for Bell’s palsy, while targeted treatments are necessary for other causes [7, 21]. Surgical options are considered for complete nerve lesions where conservative treatment fails, utilizing nerve or muscle transfers to restore facial movements. Accurate assessment is essential for guiding effective treatment. This systematic review aims to identify and validate the most effective assessment tools and scales for evaluating the severity and disability associated with PFP, updating the evidence base and including innovative assessment methods. By doing so, it seeks to enhance the accuracy of assessments, improve patient outcomes, and inform targeted and effective rehabilitation strategies [22–25]. This review will address the gap left by previous reviews, which primarily focused on impairment measures and excluded tools requiring specialized equipment, by encompassing both traditional and advanced assessment instruments and their validation in clinical practice [11, 26].

## Materials and methods

This systematic review was carried out following the methodological guidance contained in the PRISMA Checklist [27].

The protocol was published in PROSPERO (International Prospective Register of Systematic Reviews) under registration number CRD42024538346. This review includes both analytical observational studies and one non-randomized controlled trial (nonRCT). Although no temporal restrictions were imposed initially, the studies included were conducted up to April 2024.

### Eligibility criteria

Eligibility Criteria: This systematic review focuses on both assessment tools and patient-reported outcome measures (PROMS) for peripheral facial paralysis. Included studies are analytical observational studies and one non-randomized controlled trial (nonRCT) that validate these instruments. Eligible studies focus on instruments evaluating one or more domains of the International Classification of Functioning, Disability, and Health (ICF), specifically:

- Structure and functions → impairment.
- Activities → disability or activity limitation.
- Participation → participation restriction.

Studies must provide a clear and comprehensive description of the assessment tool or scale and the methods of administration. Included studies target an adult population with unilateral peripheral facial paralysis of any etiology, with no restrictions on sex or ethnicity. This review imposes no temporal or geographical restrictions. Studies not meeting these eligibility criteria were excluded.

### Information sources

The literature search was conducted across the following databases: PubMed, CINAHL, Scopus, and Web of Science. Searches were performed between July and September 2023.

### Search strategy

The clinical question was formulated using the PI(R)O method:

- P (Population): Patients with peripheral facial paralysis of the seventh cranial nerve.
- I (Intervention): Scales and instruments for assessing severity and disability.
- R (Reference standard): Potential comparison scales (e.g., House-Brackmann, Sunnybrook).
- O (Outcome): Accuracy, validity.

Given the absence of a gold standard, included studies compare the assessed scale with commonly used scales such as House-Brackmann, Sunnybrook, or the Facial Disability Index (FDI). The keywords for the search were derived from the PI(R)O framework. For PubMed, the search utilized MeSH terms to increase specificity, resulting in the following search string:

((“Facial Paralysis“[MeSH] OR “Bell Palsy“[MeSH] OR “Facial Nerve Diseases“[MeSH] OR “Facial Muscle“[MeSH] OR “Facial Expression“[MeSH] OR “Facial Injuries“[MeSH] OR “Orofacial Disorders“[MeSH]) AND (“Assessment“[MeSH] OR “Evaluation“[MeSH] OR “Validation“[MeSH] OR “Diagnostic Techniques and Procedures“[MeSH] OR “Outcome Assessment (Health Care)“[MeSH] OR “Patient Outcome Assessment“[MeSH] OR “Scales“[MeSH] OR “Questionnaires“[MeSH] OR “Patient Reported Outcome Measures“[MeSH] OR “PROMS” OR “Reliability“[MeSH] OR “Validity“[MeSH] OR “Psychometrics“[MeSH]) AND (“Orofacial Assessment” OR “Facial Motor Skills” OR “Orofacial Myofunctional” OR “Facial Palsy” OR “Synkinesis” OR “Facial Asymmetry” OR “Facial Disability” OR “Facial Function” OR “Facial Movement” OR “House-Brackmann” OR “Sunnybrook” OR “Facial Grading Systems” OR “Facial Assessment Tools” OR “Facial Nerve Grading” OR “Facial Paralysis Assessment” OR “Facial Paralysis Rehabilitation”))

For other databases, simple and advanced searches were conducted using the following keywords:

- Bell palsy, Facial paralysis, facial palsy.
- Grading system, assessment, tool, evaluation, scale.
- Instrument validation, validity.

### Study selection

The study selection process was conducted by two independent reviewers to ensure the reliability and accuracy of the inclusion criteria. The steps involved were:

Identification: Total studies identified through search strings across different databases were recorded. Duplicates were removed using Zotero.

Screening: Studies were excluded based on title relevance.

Eligibility: Articles not meeting the inclusion criteria were excluded after reading the abstract and, if necessary, the full text.

Inclusion: Remaining studies were selected for the systematic review.

**Fig. 1:**
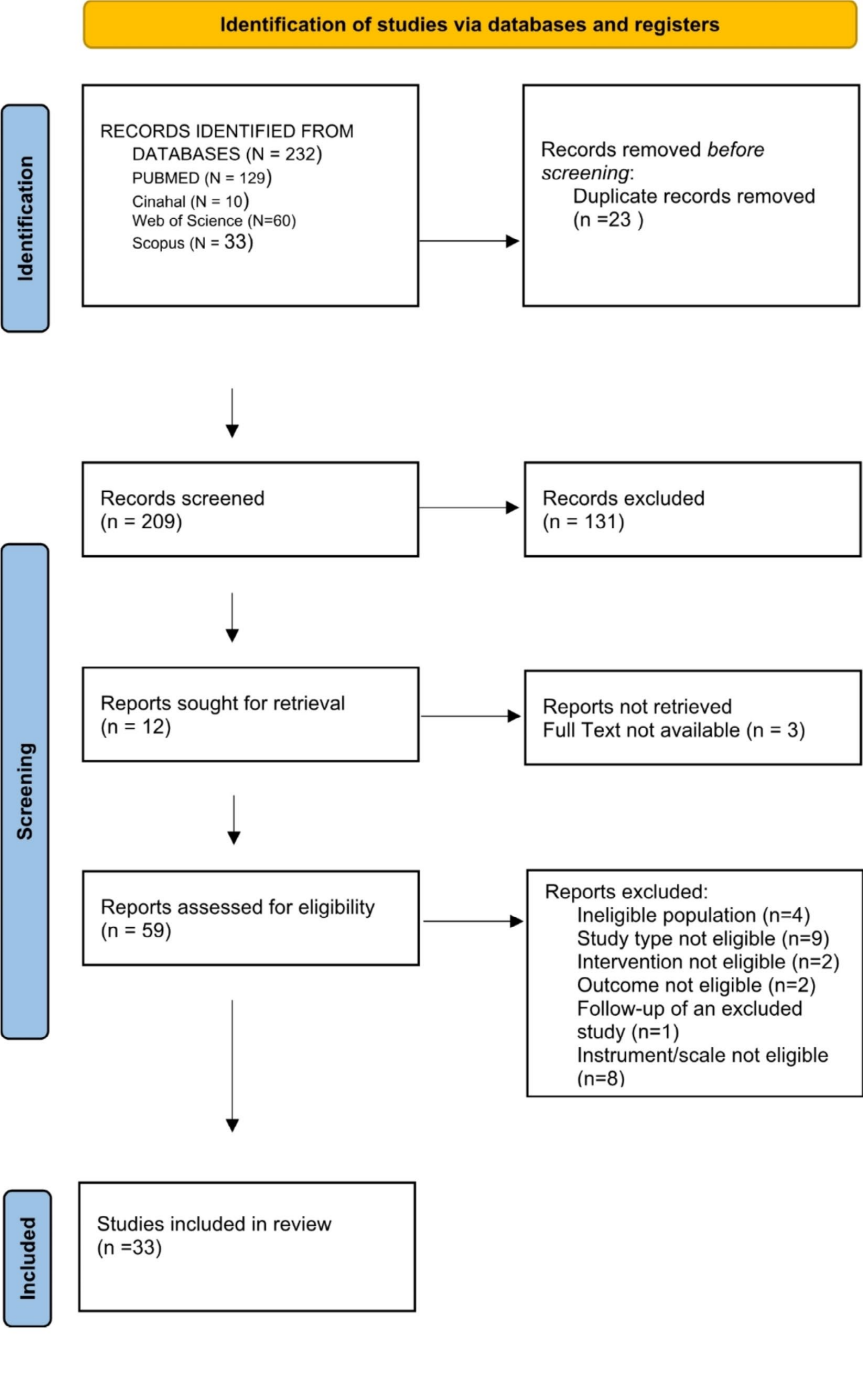
PRISMA flow-diagram

The selection process was summarized using the PRISMA 2020 Flow Diagram (Fig. 1).

### Data collection and data items

Data extraction was performed by a single reviewer through careful reading of each included article. Extracted data included:

- Title and authors.
- Year of publication.
- Country of origin.
- Study design.
- Sample size.
- Mean age.
- Inclusion criteria.
- Assessment tool evaluated.
- Reference standards/scales used for comparison.
- Validation results (validity, reproducibility, correlation with other scales).

### Methodological quality and risk of bias assessment

The methodological quality and risk of bias for each selected study were assessed using the CASP (Critical Appraisal Skills Programme) checklist [28]. The CASP checklist provides a systematic approach to critically appraise research studies by evaluating their validity, results, and relevance. It includes sections such as the validity of study results, the reliability of the findings, and their applicability to clinical practice. For this review, the CASP checklist ensured comprehensive evaluation, focusing on key aspects like study design, population description, and methodology.The checklist items are divided into three sections:

- **Section A**: Are the study results valid?
- **Section B**: What are the results?
- **Section C**: Will the results help locally?

The methodological quality of each study was evaluated using the relevant applicable items, ensuring a comprehensive assessment of their validity and applicability in clinical practice (Table 1).

## Results

From the search conducted in electronic databases, which concluded in September 2023, a total of 232 results were obtained: 129 from PubMed, 10 from CINAHL, 60 from Web of Science, and 33 from Scopus. After removing 23 duplicates using Zotero, 209 articles remained. Following title and abstract screening, 139 articles were excluded. Twelve articles were sought for retrieval, but three were not available in full text. Detailed reasons for the exclusion of full-text articles were based on criteria such as study design, intervention type, and outcome relevance. The remaining 67 articles were read in full, and 33 were included in the systematic review(Fig. 1).

**Table 1:** Characteristics of the included studies

Authors and Year of Publication	Evaluated Instrument	Comparison Instrument	Sample Size and Characteristics	Etiology, Rehabilitation, and Surgery	Description	Results
Ross et al. (1996)[12]	Facial Grading System (FGS) or Sunnybrook (SB)	House-Brackmann facial grading system (HB)	19 patients with unilateral facial paralysis	Bell’s palsy, trauma; Physical therapy (neuromuscular retraining, exercises to improve facial muscle coordination); No surgical intervention	Patient videos pre and post-treatment were analyzed once by a single evaluator.	The scale was found to be valid and sensitive.
VanSwearingen et al. (1996)[13]	The Facial Disability Index	FGS; The Primary Care Evaluation of Mental Disorders screening tool	46 patients (30 females, 16 males); mean age: 46.8 years (SD = 15.6)		The questionnaire was administered once, along with the comparison scales.	Internal consistency showed a theta coefficient > 0.8. There was a correlation with the comparison scales (*r* = 0.066 for FGS and 0.694 for the Primary Care Evaluation of Mental Disorders screening tool).
Yuen et al. (1997)[14]	Moiré Topography	HB	51 patients with unilateral facial paralysis and 10 healthy volunteers; mean age: 45.3 years	Bell’s palsy, surgical injury; Physical therapy (exercises, manual therapy), psychological support; Some had facial nerve decompression surgery	Photos of patients were analyzed by two expert physicians.	Strong correlation with the HB scale.
Smith et al. (1992)[29]	HB	Scales by Botman and Jongkees, May, Pietersen, Smith, Yanagihara, Stennert, Adour, and Swanson	10 patients with facial paralysis for at least 1 year	Bell’s palsy, trauma; Physical therapy (facial exercises, electrical stimulation); No surgical intervention	Patients were evaluated by 4 clinicians with varying experience using all scales. Presentation order to clinicians was random.	Reproducibility was considered acceptable (K value = 0.642–0.768).
Kahn et al. (2001)[30]	The Facial Clinimetric Evaluation Scale (FaCE Scale)	HB; FGS	86 subjects including patients with PFP and a healthy control group (55 females, 31 males); mean age: 50.5 years	Various etiologies; Physical therapy (customized exercise programs); No surgical intervention	The scale was administered twice, two weeks apart. In a subgroup of 41 patients, the HB and SB scales were also administered.	Internal consistency had a Cronbach’s alpha > 0.70. Test-retest correlation was 0.88. Statistically significant correlation with HB, FGS, and FDI scales.
Coulson et al. (2005)[31]	Sunnybrook	Sydney Scale; HB	21 patients (11 females, 10 males) with PFP; mean age: 47.7 years	Bell’s palsy, idiopathic, trauma; Physical therapy (facial muscle strengthening exercises); No surgical intervention	Evaluation performed independently by six otolaryngologists twice, with random order of patients.	Fair reliability of the SB scale with a mean ICC = 0.63 ± 0.23 for “voluntary movements” and “synkinesis.” Correlation with the Sydney scale was *r* = 0.79 for voluntary movements and 0.36 for synkinesis.
Hu et al. (2001)[32]	Sunnybrook	No	2 patients and 2 healthy subjects (control group)	Bell’s palsy, trauma; Physical therapy (neuromuscular retraining, facial massage); No surgical intervention	Evaluation performed by eight novices twice, three weeks apart.	Intra-operator ICC ranged from 0.838 to 0.929; inter-operator ICC was 0.982 for the first evaluation and 0.970 for the second.
Lazarini et al. (2006)[33]	HB (graphical adaptation version)	HB (original version)	32 patients with PFP	Bell’s palsy; Physical therapy (facial muscle exercises, functional retraining); No surgical intervention	Evaluation performed independently by three clinicians first with the HB scale, then with the graphical version after one week, and after 30 days with both scales.	Inter-rater agreement expressed by concordance index was 40.6, higher than the original version’s 37.5.
Manktelow et al. (2008)[34]	Ruler Measurement	Electronic Instrumentation	21 adult patients with PFP and 10 healthy subjects	Bell’s palsy; Physical therapy (manual therapy, neuromuscular exercises); No surgical intervention	Evaluation performed on all participants by two surgeons twice on the same day.	Mean ICC for inter-operator reproducibility was 0.879 for static measurements and 0.935 for dynamic measurements. Intra-operator ICC values were > 0.789.
Vrabec et al. (2009)[35]	Facial Nerve Grading Scale 2.0	HB	21 patients	Bell’s palsy, trauma; Physical therapy (neuromuscular retraining, facial exercises); No surgical intervention	Videos were analyzed by 14 clinicians with both scales.	Inter-operator reproducibility K value was 0.395, mean ICC = 0.986, concordance rate was 67.9%.
de Almeida et al. (2010)[36]	Lip Reanimation Outcomes Questionnaire (LROQ)	No	19 patients (10 males, 9 females); mean age: 63.2 years with PFP undergoing lip reanimation surgery	Bell’s palsy; Physical therapy (muscle coordination exercises, manual therapy); No surgical intervention	Evaluation by 6 examiners with varying experience.	Internal consistency was excellent for both parts of the questionnaire (Cronbach’s alpha range = 0.813–0.915 for the first part, 0.736 for the second part). Test-retest ICC range was 0.616–0.981.
Reitzen et al. (2009)[37]	HB (regional assessment)	HB (original version)	11 patients (5 females, 6 males); mean age: 60 years	Post-surgical (lip reanimation); Physical therapy (post-surgical rehabilitation, muscle strengthening); Lip reanimation surgery performed	Evaluation performed by 14 clinicians/students.	Inter-operator reproducibility K values ranged from 0.469 to 0.597 among the more experienced evaluators.
Kanerva et al. (2006)[38]	Sunnybrook	HB	8 patients with PFP	Bell’s palsy, trauma; Physical therapy (manual therapy, facial exercises); No surgical intervention	Evaluation performed by 28 examiners twice, three weeks apart.	Intra-operator ICC for the Sunnybrook scale ranged from 0.864 to 0.995; inter-operator ICC was 0.997.
Mehta et al. (2007)[39]	Synkinesis Assessment Questionnaire	FGS (synkinesis part)	65 patients with PFP (48 females, 17 males); mean age: 46.8 years and 20 healthy subjects	Bell’s palsy; Physical therapy (facial muscle exercises, coordination training); No surgical intervention	Questionnaire administered to 28 patients twice, three weeks apart; 37 patients before and after botulinum toxin treatment. FGS administered to a subgroup of 39 patients.	Internal consistency of the questionnaire was acceptable (Cronbach’s alpha = 0.683), test-retest coefficient r was 0.881. Sensitive to treatment (mean pre-treatment score = 43.6, mean post-treatment score = 25.7).
Neely et al. (2010)[40]	Sunnybrook	HB	30 patients with PFP	Bell’s palsy, trauma; Physical therapy (neuromuscular retraining, facial exercises); Some received botulinum toxin injections	Evaluated by 2 examiners in 4 sessions.	Intra-operator ICC = 0.976 and 0.958; inter-operator ICC = 0.927.
Alicandri-Ciufelli et al. (2013)[41]	The Rough Grading System (RGS)	HB	50 patients with PFP (22 males, 28 females); mean age: 54 years	Bell’s palsy, idiopathic; Physical therapy (muscle coordination exercises, neuromuscular retraining); No surgical intervention	Evaluations performed by two independent groups of clinicians (one using the RGS scale and the other using the HB scale).	Inter-operator reproducibility had a mean ICC value of 0.59. Correlation with the HB scale had r values ranging from 0.86 to 0.90.
Kecskés et al. (2011)[42]	Glasgow Facial Palsy Scale	HB, Yanagihara, Sunnybrook, Stennert-Limberg Frentrup Scale	40 patients (28 females, 12 males); mean age: 52 years	Bell’s palsy, trauma; Physical therapy (facial exercises, muscle strengthening); No surgical intervention	Evaluations performed independently by 3 clinicians.	Strong correlation with comparison scales (*r* > 0.64).
Banks et al. (2015)[43]	eFACE	No	25 patients (60% females, 40% males); mean age: 55 years	Bell’s palsy, trauma; Physical therapy (neuromuscular exercises, manual therapy); No surgical intervention	Two expert clinicians evaluated the participants twice, two weeks apart.	Inter-operator reproducibility had an ICC = 0.97. Intra-operator reproducibility showed no significant differences between evaluations.
Tzou et al. (2015)[44]	Chaung’s Smile Excursion Score	No	34 patients	Bell’s palsy, trauma; Physical therapy (facial muscle exercises, functional retraining); No surgical intervention	Evaluated by three independent raters with the proposed tool four times, two days apart.	Inter-operator reproducibility had a mean ICC value of 0.948; intra-operator ICC values were 0.989, 0.991, and 0.999.
Pavese et al. (2013)[45]	Sunnybrook (Italian version)	No	23 patients (10 males, 13 females); mean age: 47 years	Bell’s palsy, trauma; Physical therapy (facial exercises, muscle coordination); No surgical intervention	Evaluations with the scale were performed by six clinicians twice, 30 days apart.	Internal consistency was high (Cronbach’s alpha = 0.91); intra-operator and inter-operator reproducibility was excellent (ICC = 0.96 and 0.98).
Pavese et al. (2014)[46]	The Facial Disability Index (Italian version)	SB, 12-item short-form health survey	100 patients with PFP	Bell’s palsy, trauma; Physical therapy (neuromuscular retraining, facial exercises); No surgical intervention	Questionnaire administered to all participants. Re-administered to 37 patients at the end of the evaluation and to 41 patients one month later along with the SB scale.	Reproducibility was excellent (ICC = 0.93 for “social function” and 0.84 for “physical function”). Internal consistency had theta coefficients of 0.82 and 0.78. Correlation with the SB scale was *r* = 0.44 and with the 12-item short-form health survey was *r* = 0.55.
Gordon et al. (2012)[47]	HB	No	40 photos of patients operated for acoustic neuroma removal	Bell’s palsy, trauma; Physical therapy (muscle strengthening, psychological support); No surgical intervention	Evaluated by 8 surgeons using the HB scale.	Internal consistency was excellent (Cronbach’s alpha = 0.97); inter-observer agreement was substantial (Kendall’s coefficient = 0.76, *p* < 0.001).
Volk et al. (2019)[48]	Video tutorial Sunnybrook	Standard video	18 patients (12 females, 6 males); mean age: 54 years	Post-surgical (acoustic neuroma removal); Physical therapy (post-surgical rehabilitation, facial exercises); No additional surgical intervention	Videos analyzed by five novices and five experts twice within an hour.	Mean ICC for intra-operator reproducibility was 0.967; mean ICC for inter-operator reproducibility was 0.921.
Lee et al. (2020)[49]	A-FPG	HB, Sunnybrook	128 patients with PFP and 2 healthy subjects	Bell’s palsy, trauma; Physical therapy (neuromuscular retraining, facial exercises); No surgical intervention	Evaluations performed by two experienced clinicians.	Good correlation with HB and SB scales (*r* = 0.738 and 0.905, respectively).
Bansal et al. (2020)[50]	Caliper Measurement	FNGS 2.0	51 patients with facial paralysis and 10 healthy subjects (control group)	Bell’s palsy, trauma; Physical therapy (muscle strengthening, coordination exercises); No surgical intervention	Evaluations performed by a single examiner.	Measurement method had good correlation with the FNGS 2.0 scale.
Malhotra et al. (2016)[51]	CADS	No	30 patients (19 females, 11 males); mean age: 60 years	Bell’s palsy, trauma; Physical therapy (facial exercises, neuromuscular retraining); No surgical intervention	Evaluated by two clinicians independently, blinded to each other’s results.	Inter-operator agreement was high: 86.7% for “cornea,” 93.3% for “resting asymmetry,” 93.3% for “dynamic function,” and 86.7% for “synkinesis.”
Malm et al. (2018)[52]	Facial Asymmetry Index (FAI)	No	40 patients with facial paralysis	Bell’s palsy, trauma; Physical therapy (muscle strengthening, manual therapy); No surgical intervention	Evaluated independently by three raters three times, 48 h apart.	Inter-operator reproducibility ICC was 0.901; intra-operator ICC values were 0.888, 0.918, 0.924.
Silva et al. (2018)[53]	Psychosocial Scale of Facial Appearance	HB, Hospital Anxiety and Depression Scale	38 patients with PFP; mean age 47.6 years	Bell’s palsy, trauma; Physical therapy (neuromuscular exercises, facial exercises); No surgical intervention	Scale administered once.	Internal consistency had Cronbach’s alpha values of 0.90. Significant correlation with comparison scales.
Van Veen et al. (2020)[54]	Stereophotogrammetry	No	60 participants (30 with PFP, 30 healthy)	Bell’s palsy, trauma; Physical therapy (muscle coordination, manual therapy); No surgical intervention	Evaluations performed independently by two examiners twice.	Inter-operator reproducibility was good (ICC range = 0.442–0.929); intra-operator ICC values were > 0.700.
Ojha et al. (2022)[55]	FAME Scale	FNGS 2.0	110 patients with Bell’s palsy (age > 18 years)	Bell’s palsy, trauma; Physical therapy (facial exercises, muscle strengthening); No surgical intervention	Evaluations performed twice with a minimum interval of 3 weeks.	Intra-operator and inter-operator reproducibility had k values > 0.9; correlation with the FNGS 2.0 scale had r values > 0.7.
Cabrol et al. (2021)[56]	Sunnybrook	HB	20 patients with PFP	Bell’s palsy, trauma; Physical therapy (neuromuscular retraining, facial exercises); No surgical interventio	Patient videos evaluated by 31 clinicians with both scales twice, 2 months apart.	Intra-operator reproducibility was almost perfect with a mean ICC = 0.915; inter-operator reproducibility had an ICC value of 0.847.
Kim et al. (2023)[57]	Emotrics	HB	23 patients (11 females, 12 males); mean age: 49.4 years	Bell’s palsy, trauma; Physical therapy (muscle strengthening, coordination training); No surgical intervention	Evaluations performed twice with both instruments.	ICC values for intra- and inter-operator reproducibility were generally > 0.80.
Ten Harkel et al. (2023)[58]	Automatic SB	SB (original version)	116 patients with PFP (49 males, 67 females); mean age: 56 years	Bell’s palsy, trauma; Physical therapy (muscle strengthening, coordination training); No surgical intervention	Evaluation with the proposed instrument compared with that performed by three expert clinicians using the SB scale.	Similar reproducibility to the original version. ICC for total score was 0.87, for resting symmetry 0.45, for voluntary movements 0.89, and for synkinesis 0.77.

Legend A-FPG: Automated Facial Paralysis Grading, CADS: Composite Autonomic Dysfunction Score, eFACE: Electronic Facial Paralysis Assessment, FAI: Facial Asymmetry Index, FAME: Facial Assessment by Morphological Examination, FGS: Facial Grading System, FNGS: Facial Nerve Grading System, HB: House-Brackmann, ICC: Intraclass Correlation Coefficient, LROQ: Lip Reanimation Outcomes Questionnaire, PFP: Peripheral Facial Paralysis, RGS: Rough Grading System, SB: Sunnybrook

The systematic review includes observational analytical studies (both prospective and cross-sectional) and one non-randomized controlled clinical trial. Four studies were published before 2000 [12–14, 29]; four between 2001 and 2005 [30–32]; eight between 2006 and 2010 [33–40] ; seven between 2011 and 2015 [41–47]; six between 2016 and 2020 [48–54]; and four between 2021 and 2023 [55–58].

Ten studies originated from the United States; three from Italy; four from Canada; two from Brazil ; two from India; two from the Netherlands; one from Japan; two from the UK; two from Korea. Additionally, one study each was conducted in Taiwan, Hungary, France, Finland, and Australia .

The number of participants ranged from a minimum of 8 to a maximum of 128 subjects, with ages spanning from 18 to 88 years. Female participants outnumbered male participants across the included studies (Table 2).

Due to the lack of a gold standard reference, most studies used other scales or assessment tools for comparison. Eleven studies did not use a comparative scale. The remaining studies employed one or more of the following scales: House-Brackmann scale, Sunnybrook scale, Yanagihara Grading System, Stennert-Limberg Frentrup Scale, and Fisch grading system.

### Sunnybrook scale (SB)

Nine studies included the Sunnybrook scale:

- Two validation studies [12, 45], one of which assessed the validity of the Italian version [45].
- Six studies evaluated intra- and inter-rater reproducibility, including four on the original scale [32, 38, 39, 56] and two on electronic versions [48, 58].
- One comparative study examined the Sunnybrook scale against the Sydney and House-Brackmann scales [31].

The Sunnybrook scale evaluates resting symmetry compared to the healthy side, facial movement symmetry, and the degree of synkinesis associated with specific facial movements. Scores are converted to a scale of 0 to 100.

The initial validation by Ross et al. [12]. demonstrated good internal validity and sensitivity. Neely et al. [40]. confirmed excellent intra- and inter-rater reproducibility (ICC = 0.970 and 0.948 for intra-rater; ICC = 0.890 for inter-rater). Hu et al. [32]. also reported high reproducibility with novices (ICC intra-rater 0.838–0.929; inter-rater 0.872–0.982). Cabrol et al. [56]. and Kanerva et al. [38]. found near-perfect reproducibility with both experts and novices. Coulson et al. [31]. highlighted moderate reliability for voluntary movements and synkinesis (ICC = 0.63 and 0.23), with high correlation for voluntary movements (*r* = 0.79) but low for synkinesis (*r* = 0.36).

Automatic versions of the Sunnybrook scale were also assessed. Ten Harkel et al. [58]. used a convolutional neural network for automatic scoring, showing reproducibility comparable to human raters (ICC = 0.87 for total score). Volk et al. [48]. demonstrated excellent reproducibility using automated video tutorials (ICC intra-rater 0.967; inter-rater 0.921). The Italian version validated by Pavese et al. [45]. showed high internal consistency (α Cronbach 0.91) and excellent reproducibility (ICC = 0.96 and 0.98).

### House-Brackmann scale (HB)

The House-Brackmann scale (HB) has six grades from I (normal function) to VI (complete paralysis). Its reproducibility in original form was deemed moderate (weighted kappa 0.73, Kanerva et al. [38]). and acceptable (k = 0.642–0.768, Smith et al. [29]). Reitzen et al. [37]. proposed a regional version with inter-rater k values from 0.469 to 0.597 among experienced clinicians. Vrabec et al. [35]. introduced FNGS 2.0, showing similar reproducibility to the original (k = 0.395, ICC = 0.986). Lazarini et al. (27) developed a graphical adaptation that improved concordance (CI = 40.6 vs. 37.5). Gordon et al. [47]. demonstrated substantial inter-observer agreement (Kendall’s coefficient 0.76, *p* < 0.001) and high internal consistency (α Cronbach 0.97).

### Rough facial nerve grading system (RGS)

The RGS, similar to the HB scale but excluding synkinesis and resting asymmetry, showed higher inter-rater reproducibility than HB (k = 0.59 vs. 0.46) and significant correlation (*r* = 0.86–0.90) in Alicandri-Ciufelli et al. [41].

### Facial motor evaluation scale (FAME)

Ojha et al. [55]. proposed the FAME scale, evaluating six facial movements with graphical representations for each severity grade. It showed high intra- and inter-rater reproducibility (k > 0.9) and good criterion validity against FNGS 2.0 (Pearson > 0.7).

#### Vernier caliper measurement

Bansal et al. [50]. assessed facial asymmetry using caliper measurements, showing good correlation with FNGS 2.0 but did not evaluate reproducibility.

#### Ruler measurement

Manktelow et al. [34]. found high reproducibility for static and dynamic measurements using a ruler (ICC inter-rater 0.879 and 0.935; intra-rater 0.789–0.933).

#### Electronic assessment tools

Emotrics: Kim et al. [57]. validated Emotrics, an automatic 2D photo analysis tool. It showed significant intra- and inter-rater reproducibility (ICC > 0.80) but variable validity against HB.

Moiré Topography: Yuen et al. [14]. demonstrated strong correlation with HB.

A-FPG: Lee et al. [49]. showed good correlation with HB and SB (*r* = 0.783 and 0.905).

Stereophotogrammetry: Van Veen et al. [54]. found acceptable reproducibility (ICC inter-rater 0.442–0.929; intra-rater > 0.700).

eFACE: Banks et al. [43]. reported reliable and reproducible results (ICC inter-rater 0.97).

Glasgow Facial Palsy Scale: Kecskés et al [42]. showed strong correlation with established scales (HB, SB, Yanagihara).

#### Post-surgical outcome measures

Lip Reanimation Outcomes Questionnaire (LROQ): Almeida et al. [36] validated LROQ, showing excellent internal consistency (α Cronbach 0.813–0.915), inter-rater reproducibility (ICC 0.852), and content validity (CVI 0.87).

Facial Asymmetry Index (FAI): Malm et al. [52]. demonstrated high sensitivity to surgical changes and excellent reproducibility (ICC inter-rater 0.901).

Chuang’s Smile Excursion Score: Tzou et al. [44]. found excellent reproducibility (ICC inter-rater 0.948; intra-rater 0.989–0.999).

#### Ocular involvement

CADS (Cornea, Static Asymmetry, Dynamic Function, Synkinesis): Malhotra et al. [51]. showed high inter-rater concordance (86.7% for cornea and synkinesis).

#### Synkinesis

Synkinesis Assessment Questionnaire (SAQ): Mehta et al. [39]. validated SAQ, showing high test-retest reliability (*r* = 0.881), acceptable internal consistency (α Cronbach 0.683), and sensitivity to treatment.

#### Disability and participation

FaCE Scale (Facial Clinimetric Evaluation Scale): Kahn et al. [30]. validated FaCE, showing high internal consistency (α Cronbach > 0.70), test-retest reliability (Spearman 0.88), and significant correlation with HB, FGS, and FDI. Pavese et al. [45] confirmed these findings for the Italian version (ICC = 0.93 for physical function; ICC = 0.84 for social function).

Psychosocial Scale of Facial Appearance (PSFA): Silva et al. [53]. validated PSFA, showing high internal consistency (α Cronbach 0.90) and significant correlation with HADS.

Facial Disability Index (FDI): VanSwearingen et al. [13]. validated FDI, showing good internal consistency (theta > 0.8) and content validity with FGS. Pavese et al. [45]. confirmed excellent reproducibility (ICC = 0.93 for physical function; ICC = 0.84 for social function) for the Italian version.

These findings collectively demonstrate the various tools’ validity and reliability for assessing facial nerve paralysis, with several instruments showing strong reproducibility and sensitivity across different clinical and research settings.

### Risk of bias

The methodological quality of each included study was evaluated using the CASP checklist. Given the absence of a gold standard for comparison, three items were deemed not applicable. Excluding these items, the overall methodological quality of the studies is generally good.

All included studies present a clearly and comprehensively formulated clinical question. Most studies provide a complete description of the included population, with the exception of nine studies, where the population is simply defined as adults with facial paralysis, lacking further details. Consequently, for these studies, item 9 (“Can the results be applied to the local population?“) is marked as “not definable.”

Five studies utilize instruments that are less applicable in daily clinical practice due to the time required, the need for qualified personnel, or the use of specialized software not yet widely available.

**Table 2:** Summary of the CASPS

Study	Clear clinical question	Comparison with reference standard	Population description	Applicability to population	Detailed methods description	Results	Reliability of results	Clinical applicability	Impact on patient management
Ross et al. (1996) [12]	Yes	Not Applicable	Incomplete	Not Definable	Yes	Yes	Yes	Yes	Yes
VanSwearingen et al. (1996) [13]	Yes	Not Applicable	Yes	Yes	Yes	Yes	Yes	Yes	Yes
Yuen et al. (1997) [14]	Yes	Not Applicable	Yes	Yes	Yes	Yes	Yes	No	No
Smith et al. (1992) [29]	Yes	Not Applicable	Yes	Yes	Yes	Yes	Yes	Yes	Yes
Kahn et al. (2001) [30]	Yes	Not Applicable	Yes	Yes	Yes	Yes	Yes	Yes	Yes
Coulson et al. (2005) [31]	Yes	Not Applicable	Yes	Yes	Yes	Yes	Yes	Yes	Yes
Hu et al. (2001) [32]	Yes	Not Applicable	Incomplete	Not Definable	Yes	Yes	Yes	Yes	Yes
Lazarini et al. (2006) [33]	Yes	Not Applicable	Incomplete	Not Definable	Yes	Yes	Yes	Yes	Yes
Manktelow et al. (2008) [34]	Yes	Not Applicable	Yes	Yes	Yes	Yes	Yes	Yes	Yes
Vrabec et al. (2009) [35]	Yes	Not Applicable	Incomplete	Not Definable	Yes	Yes	Yes	Yes	Yes
de Almeida et al. (2010) [36]	Yes	Not Applicable	Yes	Yes	Yes	Yes	Yes	Yes	Yes
Reitzen et al. (2009) [37]	Yes	Not Applicable	Yes	Yes	Yes	Yes	Yes	Yes	Yes
Kanerva et al. (2006) [38]	Yes	Not Applicable	Yes	Yes	Yes	Yes	Yes	Yes	Yes
Mehta et al. (2007) [39]	Yes	Not Applicable	Yes	Yes	Yes	Yes	Yes	Yes	Yes
Neely et al. (2010) [40]	Yes	Not Applicable	Incomplete	Not Definable	Yes	Yes	Yes	Yes	Yes
Alicandri-Ciufelli et al. (2013) [41]	Yes	Not Applicable	Yes	Yes	Yes	Yes	Yes	Yes	Yes
Kecskés et al. (2011) [42]	Yes	Not Applicable	Yes	Yes	Yes	Yes	Yes	No	No
Banks et al. (2015) [43]	Yes	Not Applicable	Yes	Yes	Yes	Yes	Yes	Yes	Yes
Tzou et al. (2015) [44]	Yes	Not Applicable	Incomplete	Not Definable	Yes	Yes	Yes	Yes	Yes
Pavese et al. (2013) [45]	Yes	Not Applicable	Yes	Yes	Yes	Yes	Yes	Yes	Yes
Pavese et al. (2014) [46]	Yes	Not Applicable	Yes	Yes	Yes	Yes	Yes	Yes	Yes
Gordon et al. (2012) [47]	Yes	Not Applicable	Yes	Yes	Yes	Yes	Yes	Yes	Yes
Volk et al. (2019) [48]	Yes	Not Applicable	Incomplete	Not Definable	Yes	Yes	Yes	No	No
Lee et al. (2020) [49]	Yes	Not Applicable	Yes	Yes	Yes	Yes	Yes	No	No
Bansal et al. (2020) [50]	Yes	Not Applicable	Yes	Yes	Yes	Yes	Yes	Yes	Yes
Malhotra et al. (2016) [51]	Yes	Not Applicable	Yes	Yes	Yes	Yes	Yes	Yes	Yes
Malm et al. (2018) [52]	Yes	Not Applicable	Yes	Yes	Yes	Yes	Yes	Yes	Yes
Silva et al. (2018) [53]	Yes	Not Applicable	Yes	Yes	Yes	Yes	Yes	Yes	Yes
Van Veen et al. (2020) [54]	Yes	Not Applicable	Yes	Yes	Yes	Yes	Yes	No	No
Ojha et al. (2022) [55]	Yes	Not Applicable	Yes	Yes	Yes	Yes	Yes	Yes	Yes
Cabrol et al. (2021) [56]	Yes	Not Applicable	Yes	Yes	Yes	Yes	Yes	Yes	Yes
Kim et al. (2023) [57]	Yes	Not Applicable	Yes	Yes	Yes	Yes	Yes	Yes	Yes
Ten Harkel et al. (2023) [58]	Yes	Not Applicable	Yes	Yes	Yes	Yes	Yes	No	No

Legend **Clear Clinical Question**: The study presents a clearly defined clinical question. **Comparison with Reference Standard**: The study includes a comparison with a reference standard. **Population Description**: The study provides a complete description of the population. **Applicability to Population**: The study results can be applied to the local population. **Detailed Methods Description**: The study provides a detailed description of the methods used. **Results**: The study presents clear and reliable results. **Reliability of Results**: The study results are reliable and free from significant bias. **Clinical Applicability**: The study methods and results are applicable in clinical practice. **Impact on Patient Management**: The study results have the potential to impact patient management and outcomes

## Discussion

The objective of this systematic review was to identify and investigate the validation of tools used in the rehabilitation of peripheral facial paralysis. The literature search revealed numerous scales, questionnaires, and electronic instruments developed to enhance the evaluative process for patients with facial paralysis. Thirty-three studies were included in the review, focusing on the validation of twenty different tools. Using the ICF categorization, seventeen of these tools pertain to the “Structure and Function” domain, while the remaining three address the “Activity and Participation” domain.

The ICF categorization of studies was relevant in distinguishing the different domains impacted by PFP. Additionally, it is important to clarify the distinction between the therapist’s judgment and Patient-Reported Outcome Measures (PROMs). The therapist’s judgment often involves clinical assessments using tools like the Sunnybrook or House-Brackmann scales, which rely on observable and measurable clinical signs. In contrast, PROMs such as the Facial Disability Index (FDI) or the FaCE Scale provide insights into the patient’s subjective experience of their condition, encompassing aspects like quality of life, social interactions, and psychological impact. Both approaches are complementary, with the therapist’s assessment providing objective measures of impairment and PROMs capturing the patient’s perspective on their disability and participation restrictions [59].

As a clinician, it is crucial to understand which assessment tools are most appropriate based on the etiology of the facial paralysis and the rehabilitation approach. For instance, the Sunnybrook and House-Brackmann scales are widely applicable across different etiologies due to their comprehensive assessment of facial movement and symmetry. However, specific surgical interventions such as XII-VII or V-VII anastomosis, or temporal lengthening myoplasty, necessitate tools that can evaluate the outcomes of these procedures. The FAME scale and the Facial Asymmetry Index (FAI) are particularly useful in these contexts as they provide detailed assessments of facial movements and symmetry. Furthermore, computerized tools like eFACE and A-FPG offer objective measurements that are valuable in postoperative evaluations and tracking progress in rehabilitation [59, 60].

The Sunnybrook and House-Brackmann scales are the most extensively studied. The Sunnybrook scale demonstrated near-perfect reproducibility for both experienced clinicians and novices [31, 32, 38, 40, 56], with minimal intra- and inter-rater variability [56]. It also showed excellent internal validity [45] and sensitivity to change [12]. The original House-Brackmann scale is easy to use but has limitations, including low inter-rater reproducibility and the inability to detect subtle facial movement differences due to its six general severity grades [29, 31, 38]. To address these issues, new versions such as the FNGS 2.0 and a visual graphic adaptation have been developed. The FNGS 2.0 offers more accurate assessments by evaluating specific facial regions and including a separate domain for synkinesis evaluation [35]. The Rough Grading System (RGS) demonstrated adequate inter-rater reproducibility but lower than the aforementioned scales. It is easy to memorize and quick to apply but does not assess resting symmetry or synkinesis, which are important for a comprehensive evaluation [41]. The FAME scale, being a graphical tool, reduces the risk of subjective scoring and has shown good validity [55]. The use of a ruler for measurement is applicable in clinical practice, being simple and equipment-free but time-consuming. Further studies are needed to investigate the reproducibility of caliper measurements [34]. Computerized programs [42, 49, 57] and specific instruments [14, 54] represent an ideal advancement for objective assessments but are currently limited by the need for time, qualified personnel, and resources. The digital application eFACE appears reliable, though further studies are required for clinical implementation [43]. Tools for assessing lip asymmetry, such as the Facial Asymmetry Index (FAI) and Chuang’s Smile Excursion Score, are valid. The FAI is a quick, objective method with excellent reproducibility [52], while the Chuang’s Smile Excursion Score is advantageous due to its simplicity and lack of need for specific training or equipment [44]. The LROQ questionnaire requires further psychometric testing on a larger population before clinical recommendation [36]. The CADS tool is the first validated instrument for assessing ocular involvement in patients with PFP [51]. The SAQ questionnaire is valid, reliable, and sensitive for synkinesis evaluation but should be used alongside clinical examination due to its reliance on patient self-assessment [39]. For disability and participation restriction assessment, the Facial Disability Index [13] and the FaCE Scale [30]are both valid questionnaires. The Psychosocial Scale of Facial Appearance needs additional studies to evaluate its stability (test-retest) [53]. Only two tools identified in this review have Italian versions: the Sunnybrook scale [45] and the Facial Disability Index [46]. The findings align partially with previous literature. The 2015 systematic review [10] concluded that the Sunnybrook scale is one of the most evaluated scales, with excellent reproducibility, internal validity, sensitivity to changes, and ease of use in clinical practice. This review confirms these findings and suggests the FAME scale as an additional tool with excellent reproducibility. Furthermore, scales evaluating specific facial regions, synkinesis, and electronic tools are valid. For disability and participation assessment, three questionnaires were identified: the Facial Disability Index, the FaCE Scale, and the Psychosocial Scale of Facial Appearance. The first two were included in the 2012 review by Ho et al. [11]. , while the latter requires further studies for reproducibility evaluation.

### Strengths of the study

This review included four databases and search engines without any exclusion criteria related to language, publication date, or country. The search strategy on PubMed was constructed using MeSH terms to increase specificity. The critical appraisal was conducted using a well-known tool in the literature, with the support of an expert physiotherapist, ensuring good methodological quality of the included studies.

### Limitations of the study

The author’s limited research experience is a potential limitation. Pilot studies or studies not focused on tool validation were excluded, raising the possibility that some scales might have been overlooked. Another limitation is the absence of a gold standard for the evaluation of peripheral facial paralysis.

### Clinical implications

The validated tools identified in this review can significantly enhance the assessment and rehabilitation of patients with peripheral facial paralysis. Clinicians should consider incorporating the Sunnybrook and House-Brackmann scales, as well as the FAME and FaCE scales, into their practice due to their demonstrated reliability and validity. The use of computerized programs and specific instruments, while promising, may require additional resources and training. Further research is necessary to validate emerging tools and ensure their applicability in diverse clinical settings.

### Protocol registration

PROSPERO (CRD42024538346)

## Conclusion

This systematic review identified and validated various tools used in the rehabilitation of peripheral facial paralysis. The Sunnybrook and House-Brackmann scales emerged as the most thoroughly studied, with the Sunnybrook scale demonstrating superior reproducibility and internal validity. Newer scales like the FAME and RGS also showed promise, with the FAME scale reducing subjectivity through its graphical format. While computerized programs and specific instruments offer objective assessments, their current application in clinical practice is limited by resource requirements. For evaluating disability and participation restrictions, the Facial Disability Index and the FaCE Scale are recommended. Continued research is essential to further validate and refine these tools, ensuring comprehensive and reliable assessment for patients with peripheral facial paralysis.

## Data Availability

No datasets were generated or analysed during the current study.
